# Nutritional value of seven demersal fish species from the North Atlantic Azores archipelago

**DOI:** 10.1016/j.fochx.2024.102046

**Published:** 2024-11-26

**Authors:** Joana Filipa Furtado Goulart, Alexandre Correia Pereira, António Manuel Barros Marques, Inês do Carmo Alves Martins

**Affiliations:** a OKEANOS- Research Unit- Faculty of Science and Technology, University of the Azores, 9901-862 Horta, Portugal; b IPMA- Portuguese Institute for Sea and Atmosphere, Av. Brasília, 1449-006 Lisbon, Portugal

**Keywords:** Demersal fish, Nutritional quality, Fatty acid composition, Fish quality, Azores archipelago, Consumers health

## Abstract

Valorization of azorean demersal fish species must focus quality. This study aims to assess the nutritional value, sodium content and fat quality index of seven commercially relevant demersal fish species from the Azores Region: blackspot seabream (*Pagellus bogaraveo*), blackbelly rosefish (*Helicolenus dactylopterus*), splendid alfonsino (*Beryx splendens*), alfonsino (*Beryx decadactylus*), forkbeard (*Phycis phycis*), offshore rockfish (*Pontinus kuhlii*) and common mora (*Mora moro*). Moisture, ash, crude protein, total sugars, total fat, fatty acid profile, sodium and salt content were assessed. B. splendends, *P. kuhlii* and *P. bogaraveo* presented the highest values for crude protein, while *P. kuhlii* and *B. decadactylus* presented the biggest value for crude fat. All species were classified as lean. Sodium and salt contents were higher in *H. dactylopterus*. This study showed the high nutritional value presented in azorean demersal fish species. Nevertheless, *B. splendens*, *H. dactylopterus*, *P. kuhlii* and *B. decadactylus* revealed higher values of lipid quality indices.

## Highlights

• Azorean demersal fish species presented high nutritional value.
• All species were classified as lean.
• *P. phycis, M. moro and P. bogaraveo* presented lowest values of lipid quality indices.
• *B. splendens, H. dactylopterus, P. kuhlii and B. decadactylus* shown superior quality.

## 1 Introduction

The quality of food products is extremely important for consumers and industries around the world and this concern has highlighted the need for greater quality control of processed and fresh food products, as well as the awareness about the importance of eating nutritiously (Matos et al., 2019). Hence, fish is gaining momentum as a result of its unique nutritional benefits representing, on a global scale, around 17 % of the total animal protein intake and its consumption has been increasing (Ahmed et al., 2022; FAO, 2022; Matos et al., 2019). Global annual per capita consumption of aquatic foods grew from an average of 9.9 kg in the 1960s to 20.5 kg in 2019. However, the rate of change across countries was highly variable with upper-middle-income countries experiencing the strongest annual growth (around 3.2 %; FAO, 2022). Portugal has one of the highest per capita consumptions of seafood, representing around 56.8 kg/capita/year, while the European average is less than half (24.9 kg/capita/year), and the Azores Region have the highest rate of fishing products consumption “per capita” (Anacleto et al., 2014; Maulvault et al., 2013).

Since the end of the 19th century, the fish fauna of the Azores islands has been studied. The former Department of Oceanography and Fisheries, of the Azores University, began regular longline fishing surveys in 1995 in order to provide data that allowed the description of the structure and spatial distribution of demersal and deep-sea fish assemblages in the Azores. As a global result, more than 460 species of marine fish have been recorded in that area (Menezes et al., 2006).

The fishing sector is an important source of income and development and the main source of human impact in the Azores sea. The bottom longline fishing model is one of the most relevant, expressed in the number of vessels, commercial landings and its economic value for the region (Diogo et al., 2015). Due to the socio-economic importance of fishing in the Autonomous Region of the Azores (RAA) and extensive demand, demersal fishing had an historic increase from 17 thousand tonnes in 1960 to 40 thousand tonnes in 1999 (ICES, 2020). Therefore, the fishing sector in the RAA significantly impacts the country's socio economic sector, making it crucial to carefully value commercially available fishery products (Gordon et al., 2003).

The target species change seasonally according to different factors such as species abundance, fishing restrictions and market demand. Some of the most commercially important demersal teleosts are the blackspot seabream (*Pagellus bogaraveo*), the blackbelly rosefish (*Helicolenus dactylopterus*), the alfonsinos (*Beryx splendens and Beryx decadactylus*), the forkbeard (*Phycis phycis*), the offshore rockfish (*Pontinus kuhlii*) and the common mora (*Mora moro*) (Santos, 2017). The vulnerability of demersal fish stocks requires a focus on product quality as opposed to mass capture. Certain commercially important species already appear to have suffered a decrease in their abundance indices, such as *Phycis phycis*, *Pontinus kuhlii*, *Conger conger*, *Beryx splendens* and *Beryx decadactylus* (Duarte et al., 2022; Santos et al., 2019). One of the approaches to valorize the quality of those fish species involves demonstrating their rich nutritional content, which is urgent given the growing demand for consumers and industry (Duarte et al., 2022).

The Mediterranean Diet is one of the healthiest eating patterns, with several studies demonstrating how this complete and balanced model can provide the right nutrients in order to promote a reduction in the mortality rate and an increase in the average life expectancy (Martins et al., 2012). This diet involves the regular consumption of fish, whose muscle contains water, proteins and lipids as main components, being a healthy and nutritious food rich in polyunsaturated fatty acids, vitamins and minerals (Marques et al., 2019; Tilami & Sampels, 2017). The proximate composition of different fish species depends on intrinsic and extrinsic factors such as genetic characteristics, habitats, food composition, eating habits, feeding rate, sex, age, tissue and size (Maulvault et al., 2011). To estimate the global nutrient content of fish muscle, it is crucial to examine its moisture content, the proportion of which is an excellent indicator of nutritional quality, as well as a quality and safety factor in food preservation (Barua et al., 2012). Fish muscle contains high quality proteins with great nutritional value as a result of the presence of all essential amino acids and because it is also highly digestible when compared to terrestrial meat proteins (Ryu et al., 2021; Tilami & Sampels, 2017). Furthermore, fish is an important source of lipids, being rich in polyunsaturated fatty acids, such as omega-3, which help maintaining good health, allowing the prevention and treatment of cardiovascular, immunological, inflammatory and neurological diseases (Li et al., 2021; Tilami & Sampels, 2017). Additionally, fish is also a source of important vitamins A, D and B complex, as well as a supplier of important dietary essential elements, such as potassium, calcium, phosphorus, iodine or selenium, being crucial for good energy, bone metabolism and normal thyroid function (Ryu et al., 2021). This mineral composition depends on the total inorganic content and the most effective technique to estimate is first to quantify the ash content of the fish (Ahmed et al., 2022).

Knowing the nutritional content of species enables classifying them based on their nutritional and functional benefits, allowing consumers to make informed decisions according to their needs.

Because it is urgent to value demersal fish species of the Azores, it is important to demonstrate their rich nutritional content, focusing on quality instead of mass capture. Therefore, the objective of this study was to evaluate and compare the nutritional and energetic value, sodium and salt content, and fat quality index of seven of the most important demersal fish species captured in the RAA.

## 2 Materials and methods

### 2.1 Sample collection and preparation

Fish specimens were collected in the Azorean Exclusive Economic Zone (EEZ), namely at Condor (38° 32′ N 29° 2′ W) and Princesa Alice (37° 48′ N, 29° 15′ W) seamounts, during the institutional annual fishing survey campaigns (not by the authors of this study), using bottom longline in different transects. Specimens were preserved on ice until arriving at the laboratory. Also, some fish specimens were purchased at the fish market and sampled immediately after purchase. Thirty specimens per species of blackbelly rosefish (*Helicolenus dactylopterus*), blackspot seabream (*Pagellus bogaraveo*), forkbeard (*Phycis phycis*), common mora (*Mora moro*), splendid alfonsino (*Beryx splendens*), alfonsino (*Beryx decadactylus*) and offshore rockfish (*Pontinus kuhlii*) were sampled. Pre-gutted fish were carefully fileted and the skin removed. Fresh muscle samples were separated to further determination of moisture and ash contents. The remaining muscle was weighted, kept at −80 °C for 24 h and freeze-dried at −60 °C for 72 h (ZIRBUS - VaCo 5) to further analysis of total fat content, crude protein, total carbohydrate, sodium content and fatty acids composition, saturated, monounsaturated and polyunsaturated.

### 2.2 Chemical analysis

Fish nutritional composition was determined by an independent and certified external laboratory (SGS Portugal S.A.). Thirty samples were pooled and four replicates from each pool were analyzed. Moisture was obtained by thermogravimetry by measuring the mass of the samples over time using a thermogravimetric analyzer (LECO, 604–100-700, TGA 701/ 105 ± 2 °C) according to NP 872, NP 875, NP 1614–1 and NP 1615 as well as ash content (LECO, 604–100-700, TGA 701/ 550 ± 20 °C) according to NP 875, NP 1614–1 and NP 1615. Crude protein determination was validated by Dumas combustion method for the determination of the total nitrogen content with a DUMAS protein analyzer (LECO, FP828) followed by a 6.25 conversion factor. Total carbohydrate content was measured by the luff schoorl method by Hight Performance Liquid Chromatography with a refractive index detector (HPLC-RID) (AGILENT TECHNOLOGIES, HPLC 1260). Total fat was determined by acid hydrolysis with solvent extraction on a Soxtec fat extractor (FOSS, Soxtec 8000) according to NP ISO 6492:2014, ISO 1735:2004, ISO 1211–2010, ISO 3727-1:2001, ISO 3727-2:2001, ISO 3727-3:2003 and ISSO 6498. Gas chromatography (GC) with a flame ionization detector (FID) was applied to quantified saturated, monounsaturated and polyunsaturated fatty acids as well as the fatty acids profile according to ISO 12966 (AGILENT TECHNOLOGIES GC-FID, 7820 A). Sodium content was measured by flame atomic absorption spectrophotometer (EAA) according to ISSO 6869, ISO 8070 IDF119 (AGILENT TECHNOLOGIES, 240AA) and salt content by calcule.

### 2.3 Nutritional value

Energy value was calculated by multiplying the relative percentage of each nutrient (protein and fat) with the correction factors, 4 kcal g^− 1^ (17 kJ g^− 1^) and 9 kcal g^− 1^ (37 kJ g^− 1^) for protein and fat, respectively, as described in the Regulation (EU) No 1169/2011, 2011.

Several different calculation methods were employed to assess the lipid quality index. Calculations were made as follows:

**Sum of SFAs (SFA):** Σ (C6:0, C8:0, C12:0, C14:0, C15:0, C16:0, C17:0, C18:0, C20:0),

**Sum of MUFAs (MUFA):** Σ (C14:1n5, C16:1n7, C17:1n7, C18:1n9t, C18:1n9c, C20:1n9, C22:1n9),

**Sum of PUFAs (PUFA):** Σ (C18:2n6t, C18:2n6c, C18:3n6, C18:3n3, C20:2n6, C20:3n3, C20:3n6, C20:4n6, C20:5n3, C22:6n3),

**Hypocholesterolemic/hypercholesterolemic ratio (h/H):** [(C18:1 + C18:2 + C18:3 + C20:3 + C20:4 + C20:5 + C22:4 + C22:5 + C22:6) / (C14:0 + C16:0)] (Duyar & Bayrakli, 2023; Fernandez et al., 2007).

**Atherogenicity Index (AI):** [C12:0 + (4 × C14:0) + C16:0] / ((n-3)PUFA + (n-6)PUFA + MUFA) (Duyar & Bayrakli, 2023; Ulbricht & Southgate, 1991).

**Thrombogenicity Index (TI):** [C14:0 + C16:0 + C18:0] / [(0.5 × MUFA) + (0.5 × (n-6)PUFA) + (3 × (n-3)PUFA) + (n-3)PUFA/(n 6)PUFA] (Duyar & Bayrakli, 2023; Ulbricht & Southgate, 1991).

**Flesh-lipid quality (FLQ):** 100 * (EPA + DHA) / total fatty acids (Abrami et al., 1992).

**Health-promoting index (HPI):** UNSAT / [C12:0 + (C14:0 × 4) + C16:0] (Chen et al., 2004).

**Polyene index (PI):** (C20:5 + C22:6) / C16:0 (Lubis & Buckle, 2007).

### 2.4 Statistical analysis

One-way analysis of variance (ANOVA) was performed to compare sample means and identify significant differences between species for nutritional composition, fatty acids content and nutritional value index. All assumptions of data normality and homogeneity of variance, required to perform ANOVA, were validated by Kolmogorov-Smirnov's and Levene tests, respectively. In post hoc multi-comparisons (*F value* and *p-value*), the Tukey test was used with the significance level of 0.05.

## 3 Results

### 3.1 Nutritional value of species

Significant differences (Tuckey test, *p* = 0.05) were found in all analyzed components of the proximate nutritional composition of fish meat from different species with the exception of moisture percentage (Table 1). Regarding moisture content, the lowest value relates to *B. splendens* (76.9 % ww) and the highest value to *M. moro* (80.2 % ww). *P. bogaraveo* and *B. decadactylus* presented the same value of 77.7 % ww of moisture. The ash content was higher in *P. bogaraveo* (2.1 % ww) and lower in *P. kuhlii* (0.9 % ww). *P.phycis* and *H. dactylopterus* presented the same value of 1.7 % ww of ash content. For crude protein *B. splendends, P. kuhlii* and *P. bogaraveo* presented the highest value (17.6 % ww) while *B. decadactylus* presented the lowest (16.1 % ww). All the analyzed species presented very low values of carbohydrates, below the detection limit of the equipment (0.1 % of wet weight). Crude fat content varied from 1.1 % ww in *M. moro* and 4.8 % ww in *P. kuhlii* and *B. decadactylus*. Sodium and Salt content were higher in *H. dactylopterus* (0.11 % ww, 0.29 % ww) and *P. bogaraveo* (0.10 % ww, 0.26 % ww) while presented lower values in *P. kuhlii* (0.05 % ww, 0.13 % ww)*.* The species with the highest energy value were *B. splendends* (109.1 Kcal 100 g^−1^) and *P. kuhlii* (113.6 Kcal 100 g^−1^), while *M. moro* presented the lowest (76.3 Kcal 100 g^−1^) value.

**Table 1:** Percentage of the nutritional parameters (moisture, ash, crude protein, total sugars, crude fat and sodium), salt and energetic value (Kcal 100 g^−1^) of species *Physis phycis* (forkbeard), *Mora moro* (common mora), *Beryx splendens* (splendid alfonsino), *Helicolenus dactylopterus* (blackbelly rosefish), *Pontinus kuhlii* (offshore rockfish), *Pagellus bogaraveo* (blackspot seabream) and *Beryx decadactylus* (alfonsino).

Component (% of wet weight)	*P. phycis*	*M. moro*	*B. splendens*	*H. dactylopterus*	*P. kuhlii*	*P. bogaraveo*	*B. decadactylus*
Moisture (%)	79.0 ± 0.5	80.2 ± 0.9	76.9 ± 1.9	78.1 ± 1.3	77.1 ± 1.6	77.7 ± 1.6	77.7 ± 2.8
Ash (%)	1.7^b^ ± 0.1	1.3^c^ ± 0.2	1.4^bc^ ± 0.2	1.7^b^ ± 0.1	0.9^d^ ± 0.2	2.1^a^ ± 0.1	1.5^bc^ ± 0.1
Crude protein (%)	16.8^ab^ ± 0.1	16.6^ab^ ± 0.1	17.6^a^ ± 0.5	16.2^b^ ± 0.2	17.6^a^ ± 0.3	17.6^a^ ± 1.0	16.1^b^ ± 0.4
Total sugars (%)	<LOD	<LOD	<LOD	<LOD	<LOD	<LOD	<LOD
Crude fat (%)	2.0^b^ ± 0.4	1.1^b^ ± 0.2	4.3^a^ ± 0.9	4.3^a^ ± 0.9	4.8^a^ ± 1.0	2.1^b^ ± 0.4	4.8^a^ ± 1.0
Sodium (%)	0.09^a^ ± 0.01	0.08^ab^ ± 0.01	0.08^ab^ ± 0.01	0.11^a^ ± 0.02	0.05^b^ ± 0.003	0.10^a^ ± 0.025	0.08^ab^ ± 0.01
Salt (%)	0.23^ab^ ± 0.03	0.21^b^ ± 0.02	0.20^bc^ ± 0.02	0.29^a^ ± 0.04	0.13^c^ ± 0.01	0.26^ab^ ± 0.06	0.21^b^ ± 0.02
Energy value (Kcal 100 g^−1^)	85.2^cd^ ± 0.4	76.3^e^ ± 0.5	109.1^a^ ± 2.2	103.5^b^ ± 0.7	113.6^a^ ± 1.5	89.3^c^ ± 4.8	83.3^d^ ± 3.0
(KJ 100 g^−1^)	356.7^cd^ ± 1.8	319.5^e^ ± 2.0	456.8^a^ ± 8.8	433.3^b^ ± 2.8	475.6^a^ ± 5.9	373.9^c^ ± 19.1	348.8^d^ ± 12.3

Values are expressed as average ± standard deviation (SD). Similar letters indicate no significant differences (Tuckey test, *p* > 0.05) among species in each row.

LOD - level of detection (0.1 % of wet weight).

The fatty acid profile of fish muscle fat was also used to characterize the nutritional value of the seven species under study (Table 2, Table 3). The most prevalent FA type in muscle fat varied between SFAs (*P. phycis, M. moro, P. bogaraveo*) and MUFAs (*B. splendends, P. kuhlii*, *B. decadactylus*), whereas PUFAs were always the least prevalent. The presence of n-3 PUFAs was above level of detection in *M. moro* while n-6 PUFAs were only identified in *B. splendends* and *P. kuhlii* with the same percentage (0.1 % ww). In all species, palmitic acid (C16:0), the sum of elaidic and oleic acids (C18:1n9t c) and the DHA (C22:6n3) were dominant in their respective groups (SFA, MUFA and PUFA, respectively).

**Table 2:** Content (% wet weight) of the main fatty acid groups (SFA, MUFA, PUFA, n-3 PUFA and n-6 PUFA) in muscle tissue of the fish species *Physis phycis* (forkbeard), *Mora moro* (common mora), *Beryx splendens* (splendid alfonsino), *Helicolenus dactylopterus* (blackbelly rosefish), *Pontinus kuhlii* (offshore rockfish), *Pagellus bogaraveo* (blackspot seabream) and *Beryx decadactylus* (alfonsino).

Component (% of wet weight)	*P. phycis*	*M. moro*	*B. splendens*	*H. dactylopterus*	*P. kuhlii*	*P. bogaraveo*	*B. decadactylus*
**SFA(s)**	1.0^b^ ± 0.2	0.6^b^ ± 0.1	1.6^a^ ± 0.2	1.7^a^ ± 0.3	1.8^a^ ± 0.3	1.0^b^ ± 0.2	2.0^a^ ± 0.3
**MUFA(s)**	0.5^b^ ± 0.1	0.4^b^ ± 0.1	1.7^a^ ± 0.3	1.7^a^ ± 0.3	2.0^a^ ± 0.3	0.8^b^ ± 0.1	2.1^a^ ± 0.3
**PUFA(s)**	0.5^cd^ ± 0.1	0.1^e^ ± 0.0	1.0^ab^ ± 0.2	1.0^ab^ ± 0.2	1.1^a^ ± 0.2	0.3^de^ ± 0.1	0.7^bc^ ± 0.1
**n-3 PUFA(s)**	0.4^c^ ± 0.1	<LOD	0.9^a^ ± 0.1	0.9^a^ ± 0.1	0.9^a^ ± 0.1	0.3^c^ ± 0.1	0.6^b^ ± 0.1
**n-6 PUFA(s)**	<LOD	<LOD	0.1 ± 0.0	<LOD	0.1 ± 0.0	<LOD	<LOD

Values are expressed as average ± standard deviation (SD). Similar letters indicate no significant differences (Tuckey test, p > 0.05) among species in each row.

LOD - level of detection (0.1 % of wet weight).

**Table 3:** Content (% wet weight) of the main fatty acid groups components (SFA(s), MUFA(s) and PUFA(s)) and nutritional value parameters (AI, TI, h/H, FLQ, HPI and PI) in muscle tissue of the fish species *Physis phycis* (forkbeard), *Mora moro* (common mora), *Beryx splendens* (splendid alfonsino), *Helicolenus dactylopterus* (blackbelly rosefish), *Pontinus kuhlii* (offshore rockfish), *Pagellus bogaraveo* (blackspot seabream) and *Beryx decadactylus* (alfonsino).

Component (% of wet weight)	***P. phycis***	***M. moro***	***B. splendens***	***H. dactylopterus***	***P. kuhlii***	***P. bogaraveo***	***B. decadactylus***
(C6:0) Caproic acid	<LOD	<LOD	<LOD	<LOD	<LOD	<LOD	<LOD
(C8:0) Caprylic acid	<LOD	<LOD	<LOD	<LOD	<LOD	<LOD	<LOD
(C12:0) Lauric acid	<LOD	<LOD	<LOD	<LOD	<LOD	<LOD	<LOD
(C14:0) Myristic acid	<LOD	<LOD	0.1 ^b^ ± 0.0	0.2^a^ ± 0.0	0.2^a^ ± 0.0	0.2^a^ ± 0.0	0.2^a^ ± 0.0
(C15:0) Pentadecanoic acid	<LOD	<LOD	<LOD	<LOD	<LOD	<LOD	<LOD
(C16:0) Palmitic Acid	0.6^b^ ± 0.1	0.3^c^ ± 0.1	0.9^a^ ± 0.1	0.9^a^ ± 0.1	1.0^a^ ± 0.2	0.5^bc^ ± 0.1	1.0^a^ ± 0.2
(C17:0) Heptadecanoic acid	<LOD	<LOD	<LOD	<LOD	<LOD	<LOD	<LOD
(C18:0) Stearic acid	0.3^a^ ± 0.1	0.2^b^ ± 0.0	0.2^b^ ± 0.0	0.2^b^ ± 0.0	0.3^a^ ± 0.1	0.2^b^ ± 0.0	0.3^a^ ± 0.1
(C20:0) Arachidic acid	<LOD	<LOD	0.2^b^ ± 0.0	<LOD	0.2^b^ ± 0.0	<LOD	0.3^a^ ± 0.1
**Total SFA**	**1.0**^**b**^ **± 0.2**	**0.6**^**b**^ **± 0.1**	**1.6**^**a**^ **± 0.2**	**1.7**^**a**^ **± 0.3**	**1.8**^**a**^ **± 0.3**	**1.0**^**b**^ **± 0.2**	**2.0**^**a**^ **± 0.3**
(C14:1n5) cis-9-Tetradecenoic acid	<LOD	<LOD	<LOD	<LOD	<LOD	<LOD	<LOD
(C16:1n7) Palmitoleic acid	<LOD	<LOD	0.2^b^ ± 0.0	0.3^a^ ± 0.0	0.2^b^ ± 0.0	0.1^c^ ± 0.0	0.2^b^ ± 0.0
(C17:1n7) cis-10-Heptadecenoic acid	<LOD	<LOD	<LOD	<LOD	<LOD	<LOD	<LOD
(C18:1n9t + C18:1n9c) Elaidic acid + Oleic acid	0.4^c^ ± 0.1	0.4^c^ ± 0.1	1.5^a^ ± 0.2	1.2^b^ ± 0.2	1.7^a^ ± 0.3	0.5^c^ ± 0.1	1.8^a^ ± 0.3
(C22:1n9) Erucic acid	<LOD	<LOD	<LOD	<LOD	<LOD	<LOD	<LOD
**Total MUFA**	**0.5**^**b**^ **± 0.1**	**0.4**^**b**^ **± 0.1**	**1.7**^**a**^ **± 0.3**	**1.7**^**a**^ **± 0.3**	**2.0**^**a**^ **± 0.3**	**0.8**^**b**^ **± 0.1**	**2.1**^**a**^ **± 0.3**
(C18:2n6c + C18:2n6t) Linoleic acid + Linolelaidic acid	<LOD	<LOD	<LOD	<LOD	<LOD	<LOD	<LOD
(C18:3n6) gamma-Linolenic acid [GLA]	<LOD	<LOD	<LOD	<LOD	<LOD	<LOD	<LOD
(C18:3n3) alfa-Linolenic acid [ALA]	<LOD	<LOD	<LOD	<LOD	<LOD	<LOD	<LOD
(C20:2n6) cis-11.14-Eicosadienoic acid	<LOD	<LOD	<LOD	<LOD	<LOD	<LOD	<LOD
(C20:3n6) cis-8.11.14-Eicosatrienoic acid [DGLA]	<LOD	<LOD	<LOD	<LOD	<LOD	<LOD	<LOD
(C20:4n6) Arachidonic acid [AA]	<LOD	<LOD	<LOD	<LOD	<LOD	<LOD	<LOD
(C20:3n3) cis-11.14.17-Eicosatrienoic acid [ETE]	<LOD	<LOD	<LOD	<LOD	<LOD	<LOD	<LOD
(C20:5n3) cis-5.8.11.14.17- Eicosapentaenoic acid [EPA]	<LOD	<LOD	0.2^a^ ± 0.0	0.1^b^ ± 0.0	0.2^a^ ± 0.0	<LOD	0.1^b^ ± 0.0
(C22:6n3) cis-4.7.10.13.16.19- Docosahexaenoic acid [DHA]	0.4^b^ ± 0.1	<LOD	0.6^a^ ± 0.1	0.7^a^ ± 0.1	0.7^a^ ± 0.1	0.2^c^ ± 0.0	0.4^b^ ± 0.1
**Total PUFA**	**0.5**^**cd**^ **± 0.1**	**0.1**^**e**^ **± 0.0**	**1.0**^**ab**^ **± 0.2**	**1.0**^**ab**^ **± 0.2**	**1.1**^**a**^ **± 0.2**	**0.3**^**de**^ **± 0.1**	**0.7**^**bc**^ **± 0.1**
Total n-3 PUFA	0.4^c^ ± 0.1	<LOD	0.9^a^ ± 0.1	0.9^a^ ± 0.1	0.9^a^ ± 0.1	0.3^c^ ± 0.1	0.6^b^ ± 0.1
EPA + DHA	0.4^b^ ± 0.1	<LOD	0.8^a^ ± 0.1	0.8^a^ ± 0.1	0.9^a^ ± 0.1	0.2^c^ ± 0.0	0.5^b^ ± 0.1
Total n-6 PUFA	<LOD	<LOD	0.1 ± 0.0	<LOD	0.1 ± 0.0	<LOD	<LOD
Total n-9 PUFA	0.4^c^ ± 0.1	0.4^c^ ± 0.1	1.5^ab^ ± 0.2	1.3^b^ ± 0.2	1.7^ab^ ± 0.3	0.6^c^ ± 0.1	1.8^a^ ± 0.3
PUFA/SFA	0.5^ab^ ± 0.1	0.2^c^ ± 0.0	0.6^a^ ± 0.2	0.6^a^ ± 0.2	0.6^a^ ± 0.2	0.3^bc^ ± 0.1	0.4^abc^ ± 0.1
MUFA/SFA	0.5^b^ ± 0.1	0.7^ab^ ± 0.1	1.1^a^ ± 0.2	1.0^a^ ± 0.3	1.1^a^ ± 0.3	0.8^ab^ ± 0.2	1.1^a^ ± 0.2
UFA/SFA	1.0^b^ ± 0.2	0.8^b^ ± 0.2	1.7^a^ ± 0.3	1.6^ab^ ± 0.3	1.7^a^ ± 0.4	1.1^ab^ ± 0.3	1.4^ab^ ± 0.3
n-6/n-3	<LOD	<LOD	0.1 ± 0.0	<LOD	0.1 ± 0.0	<LOD	<LOD
n-3/n-6	<LOD	<LOD	9.0 ± 2.1	<LOD	9.0 ± 2.1	<LOD	<LOD
AI	<LOD	<LOD	0.5 ± 0.1	<LOD	0.6 ± 0.1	<LOD	<LOD
TI	<LOD	<LOD	0.1 ± 0.0	<LOD	0.1 ± 0.0	<LOD	<LOD
h/ H	1.3^bc^ ± 0.3	1.3^bc^ ± 0.5	2.3^a^ ± 0.4	1.8^abc^ ± 0.3	2.2^ab^ ± 0.5	1.0^c^ ± 0.2	1.9^ab^ ± 0.4
FLQ	20.0^a^ ± 0.0	<LOD	18.6^b^ ± 0.0	18.2^b^ ± 0.0	18.4^b^ ± 0.0	9.5^d^ ± 0.0	10.4^c^ ± 0.0
HPI	1.7^a^ ± 0.3	1.7^a^ ± 0.4	2.1^a^ ± 0.3	1.6^a^ ± 0.2	1.7^a^ ± 0.3	0.8^b^ ± 0.1	1.6^a^ ± 0.3
PI	0.7^ab^ ± 0.1	<LOD	0.9^a^ ± 0.1	0.9^a^ ± 0.1	0.9^a^ ± 0.2	0.4^b^ ± 0.1	0.5^b^ ± 0.1

Values are expressed as average ± standard deviation (SD). Similar letters indicate no significant differences (Tuckey test, p > 0.05) among species in each row.

LOD - level of detection (0.1 % of wet weight).

Significant differences (Tuckey test, *p* = 0.05) were found in the FA composition of fish meat from different species. The fraction of SFA varied between 0.6 % ww in *M. moro* and 2 % ww in *B. decadactylus,* being that *P. phycis and P. bogaraveo* presented the same value of 1.0 % ww of SFA content. Myristic acid (C14:0) was not detected in *P. phycis* as well as in *M. moro* and presented a significantly lower value for *B. splendens* (0.1 % ww). Palmitic acid (C16:0) percentage was higher in *B. splendens* (0.9 % ww), *H. dactylopterus* (0.9 % ww)*, P. kuhlii* (1.0 % ww) and *B. decadactylus* (1.0 % ww). Stearic acid (C18:0) percentage was higher in *P. phycis, P. kuhlii* and *B. decadactylus with the same value* (0.3 % ww). Arachidic acid (C20:0) was only detected in *B. splendens* (0.2 % ww), *P. kuhlii* (0.2 % ww) and *B. decadactylus* (0.3 % ww).

The fraction of MUFA varied between 0.4 % ww in *M. moro* and 2.1 % ww in *B. decadactylus* being that *B. splendens* and *H. dactylopterus* presented the same value of 1.7 % ww of MUFA content. Palmitoleic acid (C16:1n7) was higher in *H. dactylopterus* (0.3 % ww) and under level of detection for *P. phycis* and *M. moro*. The sum of elaidic and oleic acids (C18:1n9t c) was higher in *B. splendens* (1.5 % ww), *P. kuhlii* (1.7 % ww) and *B. decadactylus* (1.8 % ww) while Eicosenoic acid (C20:1n9) was only detected in *H. dactylopterus* and *P. bogaraveo* with equal value (0.1 % ww).

The fraction of PUFA varied between 0.1 % ww in *M. moro* and 1.1 % ww in *P. kuhlii* being that *B. splendens* and *H. dactylopterus* presented the same value of 1.0 % ww of PUFA content. EPA (C20:5n3) presented higher values in *B. splendens* and *P. kuhlii with the same percentage* (0.2 % ww) and no values for *P. phycis*, *M. moro* and *P. bogaraveo*. DHA (C22:6n3) revealed a bigger presence in *B. splendens* (0.6 % ww), *H. dactylopterus* (0.7 % ww) *and P. kuhlii* (0.7 % ww) while was not detected in *M. moro*.

Based on quality indexes, significatively variations (Tuckey test, *p* = 0.05) among species were also observed (Table 3). The biggest value of 0.6 for PUFA/SFA ratio were found in *B. splendens*, *H. dactylopterus and P. kuhlii* being that *M. moro* presented the lower value of 0.2. MUFA/SFA ratio presented higher values in in *B. splendens* (1.1), *H. dactylopterus* (1.0)*, P. kuhlii* (1.1) and *B. decadactylus* (1.1). UFA/SFA ratio varied significatively between the higher values in *B. splendens* and *P. kuhlii (both* 1.7) and the lower values in *P. phycis* (1.0) and *M. moro* (0.8)*.* EPA + DHA had the higher values for *B. splendens* (0.8 % ww), *H. dactylopterus* (0.8 % ww) and *P. kuhlii* (0.9 % ww) meanwhile it got the lower value for *P. bogaraveo* (0.2 % ww) and no value for *M. moro*. The h/H calculated ratio was higher in *B. splendens* (2.3) and lower in *P. bogaraveo* (1.0). Regarding n-6/n-3 and n-3/n-6 ratios, AI and TI, it was only possible to determine them in *B. splendens and P. kuhlii* due to the under level of detection values for n-6 PUFA in the other species and there were no significant differences between them (Tuckey test, p = 0.05). Since it was not possible to determine FLQ and PI in *M. moro*, *P. bogaraveo* presented the lowest values in both indices (9.5 and 0.4, respectively) as well as in HPI (0.8). *P. phycis* presented the highest value for FLQ (20.0), *B. splendens* for HPI (2.1) and the three species *B. splendens*, *H. dactylopterus* and *P. kuhlii* presented the highest same value for PI (0.9 for the three species).

## 4 Discussion

This work shows a distinct difference of nutritional content among the studied species which has been reported as a consequence of species gender, age, capture season, gonad maturation stage, environmental parameters (especially ocean temperature) and diet (Maulvault et al., 2011). Determining moisture allows us to know the water content of the fish muscle, having a direct effect on the texture since fish with lower water content have a harder texture. In general, in raw fish muscle, the moisture content is about 79 % ww (Nogueira et al., 2013), which is consistent with our results in which the values varied between 76.9 % ww in *B. splendens* and 80.2 % ww in *M. moro* with no statistically significant difference. Several studies showed that tissue increase in water density is related with the decrease in protein and lipids contents as well as a lower energy density, which is an indicator of a fish nutritional and physiological status and associated with reduced feeding rates and improved water quality (Li et al., 2021; Nogueira et al., 2013). The *P. phycis* present a moisture content of 78.6 % that is consistent with the results found by Nogueira et al. (2013) for the same species. However, moisture results for the *P. kuhlii* (77.1 % ww) are 24 % higher than those found for the same species in Nogueira et al. (2013) study. In the present work, although there is no statistically significant correlation between moisture and protein content (*p* > 0.05; *r* = −0.23) or moisture and lipids content (p > 0.05; *r* = −0.64), a trend for a negative correlation was noticed in both cases for the *P. kuhlii* as well for the other species.

The ash content in fish muscle is representative of the total amount of minerals available for consumers. In this study, the ash content on raw muscle fish varied between 0.9 % ww in *P. kuhlii* and 2,1 % ww *P. bogaraveo* which is consistent with other studies using a wide variety of marine species that reported ranges of 0.7 % to 5.3 % ww (Bogard et al., 2015), 1.9 % to 17.8 % ww (Flowra et al., 2012) and lower than the range of 5 % to 13 % reported by Duarte et al. (2022). For the *P. phycis* ash content are 30 % higher than the results found by Nogueira et al. (2013) for the same species (1.3 % ww). In reverse, for *P. kuhlii* the ash content is 33 % lower than those found in the same study (2.75 % ww), proving that there are several internal and external factors that influence the nutritional composition of a species. Our results show higher ash percentage values for the species *P. bogaraveo*, *P. phycis* and *H. dactylopterus*, which may have a greater amount of minerals than the other species under study and therefore may be a better source of minerals for the consumer.

Fish is one of the highest protein foods and the muscle protein in fish is very digestible into several essential amino acids, by people of all ages, compared to most terrestrial meats (Ryu et al., 2021). According to Murray and Burt (2001), the finfish muscle protein content is, approximately, 18 to 22 g per 100 g of edible portion, which is very close to the results found in this study for all the species analyzed. The protein content ranged from 16.1 % ww in *B. decadactylus* to 17.6 % ww for *B. splendends, P. kuhlii* and *P. bogaraveo*. The protein content found for *P. phycis* (16.8 %) is consistent with the results found by Nogueira et al. (2013) for the same species (18.5 % ww). However, the results obtained for *P. kuhlii* are almost half of those found in the same study. These results indicate that the species captured in the Azores have a higher protein content than the same species captured in the Northeast Atlantic, witch can be related to their nutritionally rich diet favored by the water temperature.

Salt is the combination in nature between the metallic ion sodium and the chloride ion. Once in aqueous systems, salt dissociates into these two ions. For adults, WHO recommends less than 2000 mg/day of sodium (equivalent to less than 5 g/day salt) which corresponds to a poorly filled teaspoon. The species under study revealed a mean salt content around 0.22 % ww. The lowest value was found in *P. kuhlii* muscle, around 0.13 g per 100 g of tissue and the highest value was found in *H. dactylopterus,* with 0.29 g of salt per 100 of tissue. Even the highest value is far below the recommended daily intake by WHO, meaning that these fish species are good diet options regarding salt content.

The caloric value of fish is related to protein and fat content and most lean or low-fat species of fish contain less than 100 kcal per 100 g (Ariño et al., 2013). The energy value from our species ranges from *M. moro* with 76.3 Kcal per 100 g to *P. kuhlii* with 113.6 Kcal per 100 g, showing significant differences in the consumption of these species in terms of energy intake. Results are consistent with the ones obtained by Nogueira et al. (2013) for *P. phycis* (81.3 Kcal/100 g). Nevertheless, our values obtained for *P. kuhlii* are 14 % lower than those reported by the author, indicating that the spatial differentiation of this last species affected muscle protein and lipid composition. The energy values obtained reveal an added value for the consumer, as it allows to consume fewer calories (without added sugars) to satisfy the daily protein and lipid needs.

Lipids play an important physiological role in providing energy, essential fatty acids and fat-soluble nutrients that are fundamental for maintaining human health. The results obtained for lipids content split the analyzed species in two different groups: the group of species with higher fat percentage, *B. splendends* (4.3 % ww)*, H. dactylopterus* (4.3 % ww)*, P. kuhlii* (4.8 % ww) and *B. decadactylus* (4.8 % ww) and the group of species with lowest content *P. phycis* (2.0 % ww)*, M. moro* (1.1 % ww) and *P. bogaraveo* (2.1 % ww)*.* Considering Ackman (1990) classification, only species from our second group can be considered as “lean” or “low fat” fish. Nevertheless, according to other authors, all species can be considered as “lean” fish because they present less than 5 % fat (Duarte et al., 2022). In any case, the consumption of any of these species is a good ally to a healthy diet due to their low fat contribution, which is advantageous for not exceeding the AMDR (Acceptable Macronutrient Distribution Range) for adults total fat intake set at 20 to 25 % of energy (Trumbo et al., 2002). The results obtained in this study are 1.5 times bigger for *P. phycis* and 4.5 times bigger for *P. kuhlii* than those obtained by Nogueira et al. (2013), indicating that these species, captured at the RAA have a higher fat content which may be related to a greater presence of essential fatty acids, a parameter that was also studied.

Therefore, the nutritional value of fish was also determined by the fish muscle fatty acids profile since they play a crucial role in the synthesis of certain eicosanoids (prostaglandins, thromboxanes and leukotrienes) and possess important antithrombotic properties. They also reduce the risk of coronary and cardiovascular disease, and prevent cancer, diabetes, and other inflammatory and autoimmune diseases (Tilami & Sampels, 2017). Our results show that the percentage of fatty acids main groups varied significantly among the fish species. Thus, it demonstrated that *B. splendens, H. dactylopterus, P. kuhlii* and *B. decadactylus* had a lower degree of saturation as well as a higher MUFAs and PUFAs content. It was also observed that the proportion of fatty acids changed significantly between species (*p* < 0.05). Therefore, the consumption of these species has a greater advantage for human health, since unsaturated fats are healthier, helping to reduce cholesterol and improve heart health. On the other side, *P. phycis* and *P. kuhlii* results are not consistent with the results found by Nogueira et al. (2013) for the same species. Although the values obtained for SFA in P.Kuhlii are similar, all the remaining values differ. In our study, *P. kuhlii* obtained values for MUFA 2.6 times higher and for PUFA 2.3 times lower. Regarding the *P. phycis* values obtained for SFA and MUFA, they are 1.6 times higher while for PUFA they are 2.1 times lower. These results demonstrate an advantage in the consumption of these species from the Azores since a diet rich in MUFA may be more advantageous than one rich in PUFA by not reducing as much the apolipoprotein A-I (Wahrburg et al., 1992).

Lauric (C12:0) and myristic (C14:0) fatty acids are known to promote hypercholesterolemia (Fernandes et al., 2014). Lauric acid values were below the detection limits, while it was possible to determine the presence of myristic acid in 5 of the 7 species under study, demonstrating a positive factor in its consumption. Regarding the results obtained for oleic acid, they are consistent with those obtained by Mendez and Gonzalez (1997) and Huynh and Kitts (2009) who demonstrated the presence of a higher content of this fatty acid in lean fish. Oleic acid, also known as omega-9, helps in the prevention and treatment of various neurological diseases, for example, it has neuroprotective effects against transient and permanent focal cerebral ischemia, as well as global cerebral ischemia (Song et al., 2019). Its presence in considerable quantity presents another advantage to the consumption of these species, mainly *B. splendens, H. dactylopterus, P. kuhlii* and *B. decadactylus*.

Long-term consumption of n-3 series PUFAs has been shown by many clinical and epidemiological studies to be a cause of decreased risk of heart disease and hypertension, as well as a strong ally in the prevention of blood clots and protection against various types of cancer (Nogueira et al., 2013). In addition, n-3 PUFAs contribute to the normal neurological development of children and reduce the risk of dementia disorders as well as Alzheimer's symptoms in the elderly, being essential throughout human development (Khan et al., 2023). On the other hand, it is advised to avoid excessive omega-6 intake to reduce the risk of cardiovascular diseases and obesity. Our results show higher levels of n-3 fatty acids than n-6 series for all the analyzed species. The values of Σn-3 ranged from 0.3 % ww in *P. bogaraveo* to a maximum of 0.9 % ww in *B. splendens, H. dactylopterus* and *P. kuhlii*. Dietary guidelines recommend a weekly consumption of 250 to 500 mg of long-chain omega-3 fatty acids EPA + DHA in order to promote human health (Berber et al., 2024). The obtained results showed remarkably high values for EPA + DHA in *B. splendens* (800 mg/100 g)*, H. dactylopterus* (800 mg/100 g) and *P. kuhlii* (900 mg/100 g). On the other hand *M. moro* presented very low values of EPA + DHA (<0.1 % ww). It was shown that *B. splendens* and *P. kuhlii* presented higher values of both EPA and DHA while *H. dactylopterus* stood out only in DHA. The flesh-lipid quality **(**FLQ) index provides the percentage correlation between PUFA n-3 major elements (EPA + DHA) and the total lipids, meaning that high values of this index, is indicative of a high quality of the lipid source to the consumer (Abrami et al., 1992). Although the species *P. phycis*, presented a small amount of EPA + DHA per 100 g, it was the specie with the highest FLQ index (20.0) followed by *B. splendens* (18.6)*, P. kuhlii* (18.4) and *H. dactylopterus* (18.2). Significantly lower values were obtained for *B. decadactylus* (10.4) and *P. bogaraveo* (9.5).

The minimum value of recommended PUFA:SFA ratio, by the British Department of Health, is 0.45 and UFA:SFA ratio is 0.35 (Teixeira et al., 2020), meaning that values above the recommended are translated into a healthy diet, especially in cardiovascular diseases prevention. In this study, the values of PUFA:SFA ratio obtained for *M. moro* (0.2)*, P. bogaraveo* (0.3) and *B. decadactylus* (0.4) are below the recommended value, while the remaining species obtained slightly higher values of 0.5 for *P. phycis* and 0.6 for *B. splendens, P. kuhlii* and *H. dactylopterus*. However, all the seven species analyzed in study show UFA:SFA ratios well above the recommended value, ranging between 0.80 for *M. moro* and 1.70 for *P. kuhli*.

The atherogenicity indice (AI) expresses the preventive antiatherogenic effect that inhibits plaque aggregation, reducing cholesterol and phospholipid levels and thrombogenicity indice (TI) expresses the tendency to form clots in the blood vessels (Duyar & Bayrakli, 2023; Ulbricht and Southgate,1991). Lower values of AI and TI measured in seafood, the greater the amount of anti-atherogenic fatty acids present and greater the potential for preventing coronary heart disease (Bentes et al., 2009). Therefore, AI and TI indices are recommended to be under 1.0 in seafood items (Fernandes et al., 2014). In this study, AI and TI indices were possible to calculate only for 2 species, *B. splendens* (0.5; 0.1) and *P. kuhli* (0.6; 0.1) and it was found that they comply with the values  stipulated for inclusion in a healthy diet. Also, TI obtained values lower than reported by Garaffo et al. (2011) and Maulvault et al. (2011) for Atlantic marine fish species, which represents an advantage for the consumption of species from the marine areas in our study.

In terms of nutritional value, there is a direct proportionality between the h/H ratio and the PUFA content. Therefore, the higher the h/H value, the more beneficial the food will be for human health (Bentes et al., 2009; Matos et al., 2019). The obtained h/H values for species under study reported a range from 1.0 in *P. bogaraveo* to 2.3 in *B. splendens*, the latter species being significatively better for human health. These results are in accordance with the determination of the health promotion index (HPI), proposed by Chen et al. (2004) to evaluate the nutritional value of dietary fat, which presented values from 0.8 in *P. bogaraveo* to 2.1 in *B. splendens*.

## 5 Conclusion

The nutritional benefits of demersal fish consumption has been studied. Overall, the study supports the high nutritional quality of demersal fish from Azores for a healthy diet. Nonetheless, significant differences were found between species supporting that depending on the nutrient, some species can be more beneficial than others considering the consumer health point of view. For example, lower unsaturated fatty acids/saturated fatty acids ratio in the forkbeard (*P. phycis),* the common mora *(M. moro)* and the blackspot seabream (*P. bogaraveo)*, as well as lower hypocholesterolemic/hypercholesterolemic ratio, omega-3 content and absence of omega-6, indicate a lower lipid quality and consequently a weaker contribution to a good diet on healthy fat when compared to the alfonsinos (*B. splendens and B. decadactylus),* the blackbelly rosefish *(H. dactylopterus)* and the offshore rockfish *(P. kuhlii).* These last species showed a great nutritional quality supported by the higher values of eicosapentaenoic acid + docosahexaenoic acid, omega-3, unsaturated fatty acids/saturated fatty acids ratio and hypocholesterolemic/hypercholesterolemic ratio.

This study brings to our knowledge the nutritional potential of these seven demersal species, with high commercial value. The high demand of fish for a healthy and high-quality protein source to human nutrition brings us the need to study the nutritional impact of the unknown demersal species that are now part of our diet.

Future studies with these species should focus on aminoacid, macro, trace and toxic elements, and vitamins to enable a comprehensive risk-benefit assessment of their consumption. Furthermore, information is needed to assess the effect of commonly applied culinary methods on the nutritional quality of those fish species' muscle, and to understand the bioaccessibility and bioavailability of the most relevant nutrients in these food matrices.

## Authors contribution

Conceived and designed experiment: JG, IM, AM. Performed experiment: JG, IM. Processed the samples: JG, AP. Analyzed data: JG, IM. All authors contributed to the writing of the paper. All authors contributed to the article and approved the submitted version.

## CRediT authorship contribution statement

**Joana Filipa Furtado Goulart:** Writing – review & editing, Writing – original draft, Visualization, Methodology, Investigation, Formal analysis, Data curation, Conceptualization. **Alexandre Correia Pereira:** Formal analysis. **António Manuel Barros Marques:** Writing – review & editing, Validation, Supervision, Methodology, Investigation, Conceptualization. **Inês do Carmo Alves Martins:** Writing – review & editing, Validation, Supervision, Resources, Project administration, Funding acquisition, Formal analysis.

## Declaration of competing interest

The authors declare that the research was conducted in the absence of any commercial or financial relationships that could be construed as a potential conflict of interest.

## Data Availability

The authors do not have permission to share data.

## Appendix A Supplementary data

Supplementary material 1 Image 1

Supplementary material 2 Image 2

Supplementary material 3 Image 3
