# Supplementary material for: Nutritional value of seven demersal fish species from the North Atlantic Azores archipelago
Source: Food Chem X. 2024 Nov 26;24:102046. doi: 10.1016/j.fochx.2024.102046 (PMC11650131; doi:10.1016/j.fochx.2024.102046)
Supplement: Supplementary file 1 — Supplementary material 1 [file mmc1.docx]

**Table 1 -** ANOVA results on differences between species and within specimens sample means of moisture, ash, crude protein, total sugars, crude fat, sodium and salt content and energetic value (Kcal 100g^-1^) in muscle tissue from the species *Physis phycis* (forkbeard), *Mora moro* (common mora), *Beryx splendens* (splendid alfonsino), *Helicolenus dactylopterus* (blackbelly rosefish), *Pontinus kuhlii* (offshore rockfish), *Pagellus bogaraveo* (blackspot seabream) and *Beryx decadactylus* (alfonsino). Significant p-values are indicated in bold.

|  | ***SS*** | ***DF*** | ***MS*** | ***F*** | ***p-value*** |
| --- | --- | --- | --- | --- | --- |
| **Moisture** |  |  |  |  |  |
| ***Between groups*** | 31.9200 | 6 | 5.3200 | 1.9275 | 0.1233 |
| ***Within groups*** | 57.9600 | 21 | 2.7600 |  |  |
| ***Total*** | 89.8800 | 27 |  |  |  |
|  |  |  |  |  |  |
| **Ash** |  |  |  |  |  |
| ***Between groups*** | 3.3943 | 6 | 0.5657 | 24.7500 | **0.0000** |
| ***Within groups*** | 0.4800 | 21 | 0.0229 |  |  |
| ***Total*** | 3.8743 | 27 |  |  |  |
|  |  |  |  |  |  |
| **Crude Protein** |  |  |  |  |  |
| ***Between groups*** | 10.7771 | 6 | 1.7962 | 8.0598 | **0.0001** |
| ***Within groups*** | 4.6800 | 21 | 0.2229 |  |  |
| ***Total*** | 15.4571 | 27 |  |  |  |
|  |  |  |  |  |  |
| **Crude Fat** |  |  |  |  |  |
| ***Between groups*** | 57.8286 | 6 | 9.6381 | 16.9514 | **0.0000** |
| ***Within groups*** | 11.9400 | 21 | 0.5686 |  |  |
| ***Total*** | 69.7686 | 27 |  |  |  |
|  |  |  |  |  |  |
| **Sodium** |  |  |  |  |  |
| ***Between groups*** | 0.0087 | 6 | 0.0014 | 7.5905 | **0.0002** |
| ***Within groups*** | 0.0040 | 21 | 0.0002 |  |  |
| ***Total*** | 0.0127 | 27 |  |  |  |
|  |  |  |  |  |  |
| **Salt** |  |  |  |  |  |
| ***Between groups*** | 0.0611 | 6 | 0.0102 | 9.6396 | **0.0000** |
| ***Within groups*** | 0.0222 | 21 | 0.0011 |  |  |
| ***Total*** | 0.0833 | 27 |  |  |  |
|  |  |  |  |  |  |
| **Energy Value (Kcal)** |  |  |  |  |  |
| ***Between groups*** | 4915.8971 | 6 | 819.3163 | 143.2729 | **0.0000** |
| ***Within groups*** | 120.0900 | 21 | 5.7186 |  |  |
| ***Total*** | 5035.9871 | 27 |  |  |  |
|  |  |  |  |  |  |
| **Energy Value (KJ)** |  |  |  |  |  |
| ***Between groups*** | 86117.0286 | 6 | 14352.8381 | 164.4701 | **0.0000** |
| ***Within groups*** | 1832.6100 | 21 | 87.2671 |  |  |
| ***Total*** | 87949.6386 | 27 |  |  |  |
|  |  |  |  |  |  |
|  |  |  |  |  |  |
|  |  |  |  |  |  |
