# Supplementary material for: Nutritional value of seven demersal fish species from the North Atlantic Azores archipelago
Source: Food Chem X. 2024 Nov 26;24:102046. doi: 10.1016/j.fochx.2024.102046 (PMC11650131; doi:10.1016/j.fochx.2024.102046)
Supplement: Supplementary file 2 — Supplementary material 2 [file mmc2.docx]

**Table 2 -** ANOVA results on differences between species and within specimens sample means of the main fatty acid groups percentage (SFA(s), MUFA(s) and PUFA(s)) in muscle tissue from the species *Physis phycis* (forkbeard), *Mora moro* (common mora), *Beryx splendens* (splendid alfonsino), *Helicolenus dactylopterus* (blackbelly rosefish), *Pontinus kuhlii* (offshore rockfish), *Pagellus bogaraveo* (blackspot seabream) and *Beryx decadactylus* (alfonsino). Significant p-values are indicated in bold.

|  | ***SS*** | ***DF*** | ***MS*** | ***F*** | ***p-value*** |
| --- | --- | --- | --- | --- | --- |
| **Total SFA** |  |  |  |  |  |
| ***Between groups*** | 6.4343 | 6 | 1.0724 | 18.8562 | **0.0000** |
| ***Within groups*** | 1.1943 | 21 | 0.0569 |  |  |
| ***Total*** | 7.6286 | 27 |  |  |  |
|  |  |  |  |  |  |
| **Total MUFA** |  |  |  |  |  |
| ***Between groups*** | 12.5943 | 6 | 2.0990 | 38.6667 | **0.0000** |
| ***Within groups*** | 1.1400 | 21 | 0.0543 |  |  |
| ***Total*** | 13.7343 | 27 |  |  |  |
|  |  |  |  |  |  |
| **Total PUFA** |  |  |  |  |  |
| ***Between groups*** | 3.5771 | 6 | 0.5962 | 29.5144 | **0.0000** |
| ***Within groups*** | 0.4242 | 21 | 0.0202 |  |  |
| ***Total*** | 4.0013 | 27 |  |  |  |
|  |  |  |  |  |  |
| **Total n-3 PUFA** |  |  |  |  |  |
| ***Between groups*** | 1.4933 | 5 | 0.2987 | 40.5430 | **0.0000** |
| ***Within groups*** | 0.1326 | 18 | 0.0074 |  |  |
| ***Total*** | 1.6259 | 23 |  |  |  |
|  |  |  |  |  |  |
|  |  |  |  |  |  |
|  |  |  |  |  |  |
|  |  |  |  |  |  |
|  |  |  |  |  |  |
|  |  |  |  |  |  |
