# Supplementary material for: Nutritional value of seven demersal fish species from the North Atlantic Azores archipelago
Source: Food Chem X. 2024 Nov 26;24:102046. doi: 10.1016/j.fochx.2024.102046 (PMC11650131; doi:10.1016/j.fochx.2024.102046)
Supplement: Supplementary file 3 — Supplementary material 3 [file mmc3.docx]

**Table 3 -** ANOVA results on differences between species and within specimens sample means of the main fatty acid groups components percentage (SFA(s), MUFA(s) and PUFA(s)) and nutritional value parameters (AI, TI, h/H, FLQ, HPI and PI) in muscle tissue from the species *Physis phycis* (forkbeard), *Mora moro* (common mora), *Beryx splendens* (splendid alfonsino), *Helicolenus dactylopterus* (blackbelly rosefish), *Pontinus kuhlii* (offshore rockfish), *Pagellus bogaraveo* (blackspot seabream) and *Beryx decadactylus* (alfonsino). Significant p-values are indicated in bold.

|  | ***SS*** | ***DF*** | ***MS*** | ***F*** | ***p-value*** |
| --- | --- | --- | --- | --- | --- |
| **Total SFA** |  |  |  |  |  |
| ***Between groups*** | 6.4343 | 6 | 1.0724 | 18.8562 | **0.0000** |
| ***Within groups*** | 1.1943 | 21 | 0.0569 |  |  |
| ***Total*** | 7.6286 | 27 |  |  |  |
|  |  |  |  |  |  |
| **Myristic Acid** |  |  |  |  |  |
| ***Between groups*** | 0.0320 | 4 | 0.0080 | 11.4286 | **0.0002** |
| ***Within groups*** | 0.0105 | 15 | 0.0007 |  |  |
| ***Total*** | 0.0425 | 19 |  |  |  |
|  |  |  |  |  |  |
| **Palmitic Acid** |  |  |  |  |  |
| ***Between groups*** | 1.8286 | 6 | 0.3048 | 18.2336 | **0.0000** |
| ***Within groups*** | 0.3510 | 21 | 0.0167 |  |  |
| ***Total*** | 2.1796 | 27 |  |  |  |
|  |  |  |  |  |  |
| **Stearic Acid** |  |  |  |  |  |
| ***Between groups*** | 0.0686 | 6 | 0.0114 | 7.2072 | **0.0003** |
| ***Within groups*** | 0.0333 | 21 | 0.0016 |  |  |
| ***Total*** | 0.1019 | 27 |  |  |  |
|  |  |  |  |  |  |
| **Arachidic Acid** |  |  |  |  |  |
| ***Between groups*** | 0.0267 | 2 | 0.0133 | 9.3023 | **0.0065** |
| ***Within groups*** | 0.0129 | 9 | 0.0014 |  |  |
| ***Total*** | 0.0396 | 11 |  |  |  |
|  |  |  |  |  |  |
| **Total MUFA** |  |  |  |  |  |
| ***Between groups*** | 12.5943 | 6 | 2.0990 | 38.6667 | **0.0000** |
| ***Within groups*** | 1.1400 | 21 | 0.0543 |  |  |
| ***Total*** | 13.7343 | 27 |  |  |  |
|  |  |  |  |  |  |
| **Palmitoleic Acid** |  |  |  |  |  |
| ***Between groups*** | 0.0800 | 4 | 0.0200 | 17.8571 | **0.0000** |
| ***Within groups*** | 0.0168 | 15 | 0.0011 |  |  |
| ***Total*** | 0.0968 | 19 |  |  |  |
|  |  |  |  |  |  |
| **Elaidic Acid + Oleic Acid** |  |  |  |  |  |
| ***Between groups*** | 9.4171 | 6 | 1.5695 | 40.1559 | **0.0000** |
| ***Within groups*** | 0.8208 | 21 | 0.0391 |  |  |
| ***Total*** | 10.2379 | 27 |  |  |  |
|  |  |  |  |  |  |
| **Total PUFA** |  |  |  |  |  |
| ***Between groups*** | 3.5771 | 6 | 0.5962 | 29.5144 | **0.0000** |
| ***Within groups*** | 0.4242 | 21 | 0.0202 |  |  |
| ***Total*** | 4.0013 | 27 |  |  |  |
|  |  |  |  |  |  |
| **Eicosapentanoic Acid [EPA]** |  |  |  |  |  |
| ***Between groups*** | 0.0400 | 3 | 0.0133 | 20.5128 | **0.0001** |
| ***Within groups*** | 0.0078 | 12 | 0.0006 |  |  |
| ***Total*** | 0.0478 | 15 |  |  |  |
|  |  |  |  |  |  |
| **Docosahexanoic Acid [DHA]** |  |  |  |  |  |
| ***Between groups*** | 0.8000 | 5 | 0.1600 | 23.7624 | **0.0000** |
| ***Within groups*** | 0.1212 | 18 | 0.0067 |  |  |
| ***Total*** | 0.9212 | 23 |  |  |  |
|  |  |  |  |  |  |
| **Total n-3 PUFA** |  |  |  |  |  |
| ***Between groups*** | 1.4933 | 5 | 0.2987 | 40.5430 | **0.0000** |
| ***Within groups*** | 0.1326 | 18 | 0.0074 |  |  |
| ***Total*** | 1.6259 | 23 |  |  |  |
|  |  |  |  |  |  |
| **EPA + DHA** |  |  |  |  |  |
| ***Between groups*** | 1.5200 | 5 | 0.3040 | 45.1485 | **0.0000** |
| ***Within groups*** | 0.1212 | 18 | 0.0067 |  |  |
| ***Total*** | 1.6412 | 23 |  |  |  |
|  |  |  |  |  |  |
| **Total n-9 PUFA** |  |  |  |  |  |
| ***Between groups*** | 9.1200 | 6 | 1.5200 | 38.8889 | **0.0000** |
| ***Within groups*** | 0.8208 | 21 | 0.0391 |  |  |
| ***Total*** | 9.9408 | 27 |  |  |  |
|  |  |  |  |  |  |
| **PUFA / SFA** |  |  |  |  |  |
| ***Between groups*** | 0.6286 | 6 | 0.1048 | 7.1966 | **0.0003** |
| ***Within groups*** | 0.3057 | 21 | 0.0146 |  |  |
| ***Total*** | 0.9343 | 27 |  |  |  |
|  |  |  |  |  |  |
| **MUFA / SFA** |  |  |  |  |  |
| ***Between groups*** | 1.3600 | 6 | 0.2267 | 5.3084 | **0.0018** |
| ***Within groups*** | 0.8967 | 21 | 0.0427 |  |  |
| ***Total*** | 2.2567 | 27 |  |  |  |
|  |  |  |  |  |  |
| **UFA / SFA** |  |  |  |  |  |
| ***Between groups*** | 3.1771 | 6 | 0.5295 | 7.0833 | **0.0003** |
| ***Within groups*** | 1.5699 | 21 | 0.0748 |  |  |
| ***Total*** | 4.7470 | 27 |  |  |  |
|  |  |  |  |  |  |
| **AI** |  |  |  |  |  |
| ***Between groups*** | 0.0288 | 1 | 0.0288 | 5.7600 | **0.0533** |
| ***Within groups*** | 0.0300 | 6 | 0.0050 |  |  |
| ***Total*** | 0.0588 | 7 |  |  |  |
|  |  |  |  |  |  |
| **TI** |  |  |  |  |  |
| ***Between groups*** | 0.0008 | 1 | 0.0008 | 1.2308 | **0.3097** |
| ***Within groups*** | 0.0039 | 6 | 0.0007 |  |  |
| ***Total*** | 0.0047 | 7 |  |  |  |
|  |  |  |  |  |  |
| **h / H** |  |  |  |  |  |
| ***Between groups*** | 5.6295 | 6 | 0.9382 | 6.0577 | **0.0008** |
| ***Within groups*** | 3.2526 | 21 | 0.1549 |  |  |
| ***Total*** | 8.8821 | 27 |  |  |  |
|  |  |  |  |  |  |
| **FLQ** |  |  |  |  |  |
| ***Between groups*** | 427.3400 | 5 | 85.4680 | 111480.0000 | **0.0000** |
| ***Within groups*** | 0.0138 | 18 | 0.0008 |  |  |
| ***Total*** | 427.3538 | 23 |  |  |  |
|  |  |  |  |  |  |
| **HPI** |  |  |  |  |  |
| ***Between groups*** | 3.6800 | 6 | 0.6133 | 7.7149 | **0.0002** |
| ***Within groups*** | 1.6695 | 21 | 0.0795 |  |  |
| ***Total*** | 5.3495 | 27 |  |  |  |
|  |  |  |  |  |  |
| **PI** |  |  |  |  |  |
| ***Between groups*** | 0.9933 | 5 | 0.1987 | 9.0715 | **0.0002** |
| ***Within groups*** | 0.3942 | 18 | 0.0219 |  |  |
| ***Total*** | 1.3875 | 23 |  |  |  |
|  |  |  |  |  |  |
